# The Research on Multi-Process Collaborative Manufacturing and Characterization Methods of Micro–Nano-Composite Layered Structures

**DOI:** 10.3390/nano15221716

**Published:** 2025-11-13

**Authors:** Shibo Xu, Shaobo Ge, Zehua Sun, Junyan Li, Ronghua Shi, Lujun Shen, Jin Zhang, Yingxue Xi

**Affiliations:** Shaanxi Province Key Laboratory of Thin Films Technology and Optical Test, School of Optoelectronic Engineering, Institute for Interdisciplinary and Innovation Research, Xi’an Technological University, Xi’an 710021, China; xushibo@st.xatu.edu.cn (S.X.);

**Keywords:** micronano structures, nano fabrication, polymer-based nanomaterials, AFM characterization, convolution effect correction

## Abstract

This paper innovatively proposes a high-precision fabrication strategy for silicon-based micro–nano-composite layered structures composed of micron-scale platforms and nanopillars, effectively addressing the challenges of alignment errors and material mismatch during manufacturing. By integrating electron beam lithography (EBL), inductively coupled plasma (ICP) etching, and ultraviolet nanoimprint lithography (NIL) into a unified multi-step workflow, the method achieves exceptional precision and efficiency in producing complex micro–nano-composite architectures. Comprehensive structural characterization is performed using scanning electron microscopy (SEM) and atomic force microscopy (AFM), with probe convolution effects carefully corrected to ensure accurate dimensional analysis. Experimental results confirm the outstanding stability and uniformity of the fabricated structures, exhibiting minimal deviations in both feature size and spatial layout. Nanopillars with diameters ranging from 50 to 200 nm are successfully integrated onto 1-µm square platforms, with the lateral deviation of 50 nm features maintained within 5% or less. Furthermore, the method effectively mitigates thermal stress-induced misalignment during the fabrication of multi-material layers, demonstrating strong potential for scalable production of advanced photonic devices and integrated nanophotonic systems. Overall, this work establishes a robust and versatile technical pathway for the precise manufacturing and quantitative characterization of micro–nano-composite structures, providing a key foundation for the next generation of photonic integration technologies.

## 1. Introduction

Micro–nano-composite structures overcome the performance limitations of single-scale architectures through the cross-scale synergistic effects between micro- and nano-scale components. They enable multi-spectral light field manipulation via multi-scale structural design and exhibit significant advantages in functional integration and application adaptability. Such structures demonstrate great potential in optical integration and photonic sensing [1,2,3]. For example, metal-based micro–nano-architectures can reconstruct optical wavefronts to replace conventional lenses, thereby breaking the diffraction limit and enhancing focusing efficiency, which offers new possibilities for integrated nanophotonic devices [4,5]. In solar energy applications, micro–nano-composite light-trapping structures effectively suppress optical reflection, extend the light absorption path, and significantly enhance solar absorption efficiency [6,7].

Micro–nano-composite structures can be generally categorized into laminated, core–shell, porous, and hierarchical configurations [8,9,10,11]. Among them, laminated structures have attracted particular attention owing to their high design flexibility and strong potential for functional integration. By carefully engineering the thickness and geometry of individual layers, micro–nano-laminated architectures can precisely control optical properties such as light focusing and absorption [12,13,14]. Therefore, this study proposes a micro–nano-laminated structure consisting of a micrometer-scale platform and a nanoscale pillar array, designed to modulate the light-field focusing intensity and enhance field localization, thereby verifying the feasibility and rationality of the proposed fabrication methodology.

Currently, the fabrication of micro–nano-laminated structures primarily follows two core technological approaches: the top-down and bottom-up methods [15,16]. The top-down approach mainly relies on lithographic techniques, where materials are precisely etched through chemical or physical processes to progressively reduce structural dimensions to the micro–nano-scale [17,18]. However, conventional lithographic fabrication typically requires multiple alignment and overlay steps, which often introduce alignment errors and substantially increase fabrication cost and complexity [19,20,21,22]. Xiaoyang Bi et al. fabricated micro–nano-composite structures composed of micrometer-scale stripes and nanoparticles using femtosecond laser technology. Although this single-step process avoided alignment errors inherent to overlay lithography, the fabrication accuracy was limited to approximately 300 nm due to the precision constraints of femtosecond laser machining [23]. Huan Wang et al. employed a multi-step self-aligned lithography process to effectively mitigate alignment errors; however, repeated exposures and electroforming required frequent sample transfers between lithography and electroplating systems, resulting in low throughput and limited scalability [24]. In contrast, the bottom-up approach—dominated by vapor deposition and related techniques—relies on the self-assembly or directed organization of atoms, molecules, or nanounits to gradually form micro–nano-composite structures [25,26,27]. Since laminated structures are typically composed of alternating materials with different thermal expansion coefficients [28,29], thermal mismatch and interfacial stress often occur during heating and cooling [30], leading to alignment deviations [31,32,33,34,35]. Jie Yang et al. fabricated a “moth-eye” micro–nano-composite structure integrated with optical thin films using plasma-enhanced chemical vapor deposition (PECVD) and inductively coupled plasma (ICP) etching [36]. Although low-temperature processing mitigated alignment issues, the process remained costly and inefficient. Therefore, this study proposes a hierarchical multi-step fabrication strategy that combines electron beam lithography (EBL) and inductively coupled plasma (ICP) etching with ultraviolet nanoimprint lithography (NIL). This integrated approach is expected to effectively address alignment challenges during fabrication while reducing cost and improving production efficiency.

In addition, scanning electron microscopy (SEM) and atomic force microscopy (AFM) are two conventional techniques used for the morphological and dimensional characterization of micro–nano-laminated structures; however, discrepancies often exist between their results [37,38,39]. SEM, as a non-contact measurement method, is particularly suitable for acquiring two-dimensional surface morphology and overall structural distribution [40,41,42]. In contrast, AFM is a contact-based technique that excels at measuring three-dimensional topographic information such as structural height [43,44,45]. Nevertheless, AFM measurements are affected by the tip-convolution effect, which introduces dimensional deviations in the acquired data [46]. Therefore, this study adopts a combined SEM–AFM characterization approach together with a tip-convolution correction method to effectively overcome the information limitations of individual techniques and eliminate morphological distortions caused by convolution effects.

To address the aforementioned challenges, this study innovatively proposes a layered multi-step collaborative fabrication process that integrates electron beam lithography (EBL) and inductively coupled plasma (ICP) etching with nanoimprint lithography (NIL). This approach enables high-precision fabrication of micro–nano-laminated structures while effectively resolving alignment issues encountered during processing. Furthermore, a combined SEM–AFM characterization technique, incorporating an AFM tip-convolution correction method, is employed to accurately extract three-dimensional morphological parameters of the fabricated structures. Collectively, this strategy provides a robust framework for the efficient fabrication and precise characterization of micro–nano-laminated architectures, thereby facilitating their applications in areas such as super-resolution imaging and optoelectronic detection, which depend on the modulation of light-field focusing intensity and enhancement of field localization.

## 2. Structure and Simulation

### 2.1. Micronano-Composite Layered Structures

To comprehensively evaluate the applicability and stability of the proposed fabrication process in complex geometries, a composite structure was designed consisting of a bottom micrometer-scale platform and an upper array of nanoscale pillars. This configuration serves primarily as a process validation model, characterized by intricate and refined morphological features that enable a detailed assessment of the fabrication performance across different scales and complex conditions.

**Figure 1:**
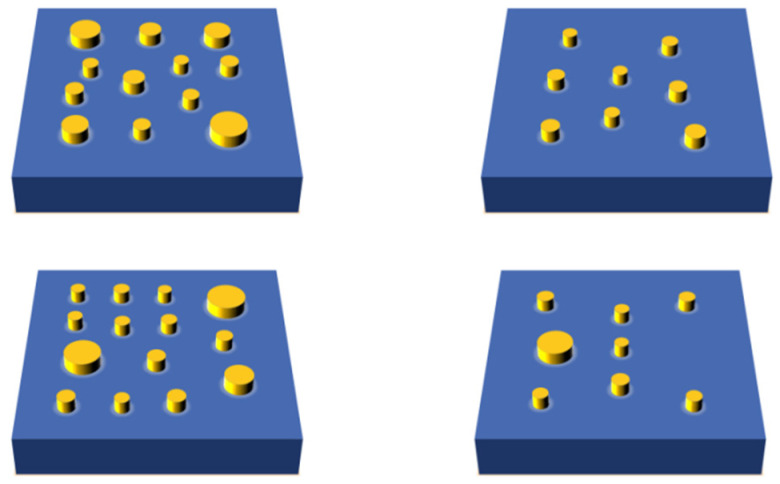
Nanopillar Structure Arrangement Schemes.

The structural material is silicon. Each unit cell comprises a micrometer-scale platform approximately 1 μm × 1 μm in size, on which nanopillars with diameters ranging from 50 nm to 200 nm are irregularly distributed. The number and spatial arrangement of these pillars were deliberately varied to increase design complexity and to thoroughly assess the process capability in terms of positional accuracy, dimensional control, and morphological uniformity. To verify process reproducibility and adaptability, four distinct nanopillar arrangement schemes, differing in distribution density, dimensional variation, and spatial configuration, were designed as shown in Figure 1. These varied designs cover a broad spectrum of fabrication difficulty levels.

### 2.2. Structural Numerical Simulation

**Figure 2:**
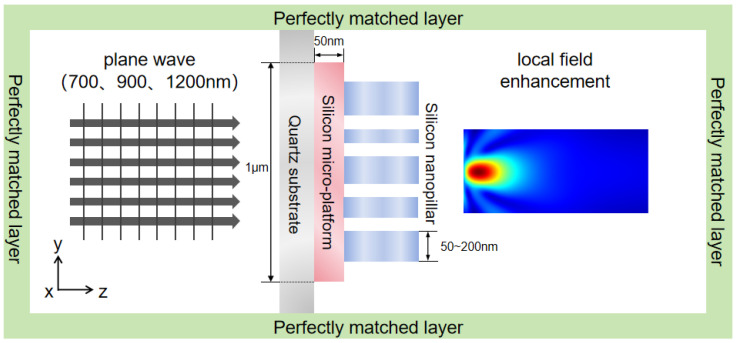
Schematic diagram of the micro–nano-composite multilayer structure with plane-wave incidence.

To verify that the proposed micro–nano-laminated structure—composed of a bottom micrometer-scale platform and a top nanoscale pillar array—can effectively modulate light-field focusing intensity and enhance field localization, a finite-difference time-domain (FDTD) simulation model was established. The model was used to analyze the light-field distribution, transmittance, and refractive characteristics of the structure under normal plane-wave incidence, thereby exploring its application potential. Each structural unit consisted of two layers: a cubic micrometer-scale base layer with a side length of 1 μm, and a metallic nanoscale pillar array positioned on top, with a thickness of 50 nm and pillar radii ranging from 50 to 200 nm. The computational domain boundaries were defined as perfectly matched layers (PMLs). Because the nanoscale layer lacked symmetry, a three-dimensional simulation model was constructed. A schematic of the simulation configuration is shown in Figure 2.

Optical performance simulations were conducted for the micro–nano-laminated structure at wavelengths of 700 nm, 900 nm, and 1200 nm to observe the light-field distribution on the x–z plane, the focusing effect on the x–y plane, and the field localization on the x–y plane.

**Figure 3:**
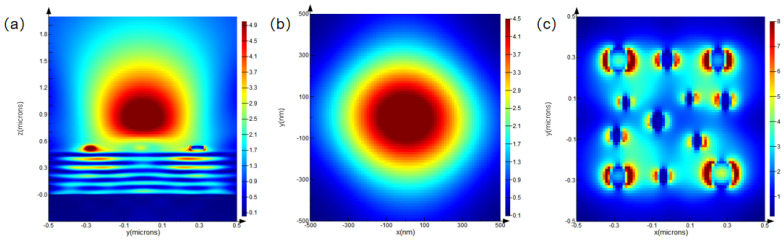
Simulated optical field distributions of the micro–nano-composite multilayer structure at a wavelength of 700 nm: (**a**) optical field distribution in the x–z plane; (**b**) focusing behavior in the x–y plane; and (**c**) localized optical field in the x–y plane.

**Figure 4:**
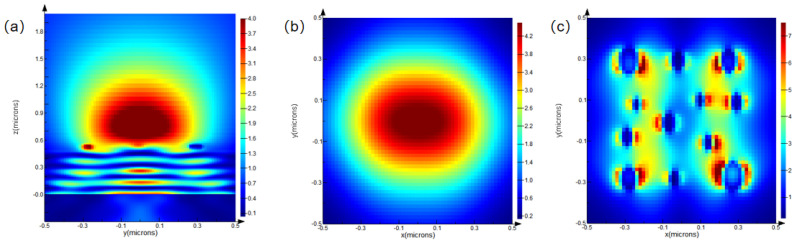
Simulated optical field distributions of the micro–nano-composite multilayer structure at a wavelength of 900 nm: (**a**) optical field distribution in the x–z plane; (**b**) focusing behavior in the x–y plane; and (**c**) localized optical field in the x–y plane.

**Figure 5:**
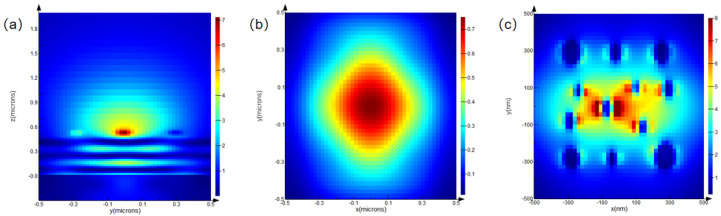
Simulated optical field distributions of the micro–nano-composite multilayer structure at a wavelength of 1200 nm: (**a**) optical field distribution in the x–z plane; (**b**) focusing behavior in the x–y plane; and (**c**) localized optical field in the x–y plane.

Analysis of Figure 3a–c, Figure 4a–c, and Figure 5a–c reveals that, at a wavelength of 700 nm, the light field in the x–z plane exhibits clear propagation characteristics and significant energy concentration. On the x–y plane, the focused light spot is circular, with highly concentrated energy, and the light field exhibits significant localization effects. At 900 nm, the energy concentration in the x–z plane changes, while, on the x–y plane, the focusing effect weakens, and the regularity and density of field localization decrease. At 1200 nm, the energy concentration in the x–z plane decreases significantly, displaying stronger divergence. On the x–y plane, the focusing ability drastically weakens, and the field localization effect deteriorates substantially.

Overall, as the wavelength increases from 700 nm to 1200 nm, the light-field energy in the x–z plane gradually decreases, the focusing ability on the x–y plane diminishes, and the degree of light-field localization declines. Optical performance is highly wavelength-sensitive, exhibiting better performance at shorter wavelengths. From the analysis above, it is evident that the micro–nano-laminated structure, composed of a micrometer-scale platform and a nanoscale pillar array, can modulate light-field focusing intensity and enhance field localization through cross-scale synergistic effects. This provides a theoretical foundation for the fabrication process and confirms the potential application of the structure in fields such as super-resolution imaging and optoelectronic detection.

## 3. Methods

### 3.1. Stepwise Processes of EBL and ICP

**Figure 6:**
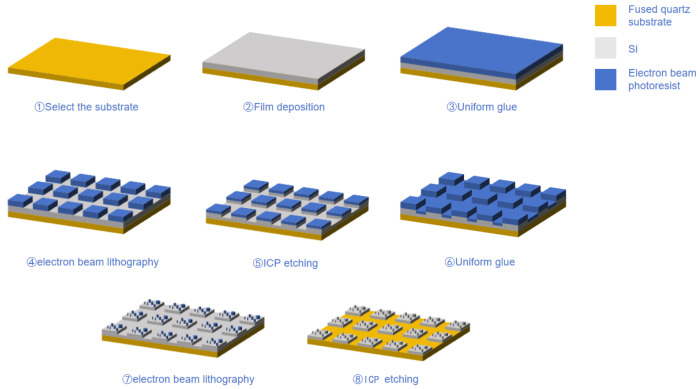
Layered Multi-Step Preparation Process for Micronano-Composite Layered Structure Master.

This study employs a layered multi-step fabrication process based on electron beam lithography (EBL) (machine model: JEOL JBX-3050MV, JEOL Ltd., Akishima, Tokyo, Japan) and inductively coupled plasma (ICP) (machine model: Plasmalab 100 ICP-RIE, Oxford Instruments Plasma Technology, Yatton, Bristol, UK)etching to achieve high-precision manufacturing of the micro–nano-laminated structure master. The fabrication process is illustrated in Figure 6. A high-flatness, double-polished fused silica substrate was used, providing a solid foundation for micro–nano-structure fabrication due to its excellent optical transparency and surface flatness [47,48,49].

First, a 200 nm thick polycrystalline silicon film was deposited onto the fused silica substrate using magnetron sputtering, serving as the etching layer for subsequent steps. The film thickness was controlled by deposition rate and time, with a tolerance of ±3%, ensuring consistency in etching depth [50]. Subsequently, PMMA (950 k A4) electron-beam resist was spin-coated onto the substrate. The spin-coating process was performed in two steps: first, at a low speed of 500 rpm for 5 s to evenly spread the resist, followed by a high-speed spin at 4000 rpm for 40 s to control the film thickness to approximately 200 nm. After spin-coating, the film was prebaked on a hot plate at 180 °C for 2 min to completely evaporate the solvent and improve adhesion, preventing bubble formation or peeling during exposure and development.

The fabrication of the micrometer-scale platform structure begins with electron beam exposure, with an acceleration voltage of 20 kV to ensure electron penetration through the resist thickness and achieve high-contrast exposure [51]. The exposure dose was controlled at 250 μC/cm^2^, balancing exposure depth and edge sharpness while preventing overexposure, which could lead to line-width enlargement. A developer solution of isopropyl alcohol and methyl isobutyl ketone (IPA:MIBK = 3:1) was used, with a developing time of approximately 60 s. After development, the sample was transferred to an ICP etching system, where a gas ratio of SF_6_ to C_4_F_8_ of 4:1 was maintained. This reacts with silicon to form volatile SiF_4_, enabling chemical etching while generating a polymer layer to suppress lateral etching and enhance etching anisotropy [52]. The etching power was set at 300 W to increase ion energy and accelerate the reaction rate. The chamber pressure was maintained at 20 mTorr to control the average free path of ions and balance etching rate with morphology stability. The process resulted in the formation of a regular 1 μm × 1 μm micrometer-scale platform array.

The fabrication of the top nanoscale pillars relies on a second round of electron beam exposure and ICP etching. After spin-coating PMMA resist again, the exposure dose was adjusted to 1000 μC/cm^2^ to improve the resolution and pattern quality of the nanoscale structures. After the development step, the sample was subjected to ICP etching, with the gas flow rates adjusted to SF_6_ at 30 sccm and C_4_F_8_ at 10 sccm. Reducing the C_4_F_8_ content weakened the formation of the passivation layer, thereby improving vertical etching efficiency. The etching power was reduced to 200 W to decrease ion bombardment energy, preventing deformation or collapse of the nanopillars. The chamber pressure was controlled at 15 mTorr to enhance ion directionality, achieving high-aspect-ratio structures. This process ensured that the nanopillar diameter ranged from 50 to 200 nm, with uniform morphology and a high-aspect ratio.

After processing, the sample was initially cleaned with isopropyl alcohol and dried, then immersed in acetone at 40 °C, and cleaned in an ultrasonic bath at 40 kHz and 50 W for 10 min to fully dissolve or peel off the resist. The sample was then transferred to isopropyl alcohol for another 5 min of ultrasonic cleaning to remove residual solvents and particles. Next, the sample was rinsed with deionized water for 1 min and finally dried with nitrogen or baked on a hot plate at 120 °C. The final micro–nano-laminated structure exhibited clear morphology and stable dimensions, meeting the design specifications.

The fabrication process parameters are shown in Table 1.

In this process, each parameter was meticulously optimized to strike a balance between etching anisotropy, surface roughness, and pattern fidelity. The control of beam dosage, gas ratio, and chamber pressure plays a crucial role in determining the final feature dimensions and aspect ratios of the micro–nano-hybrid structures.

### 3.2. NIL Process

**Figure 7:**
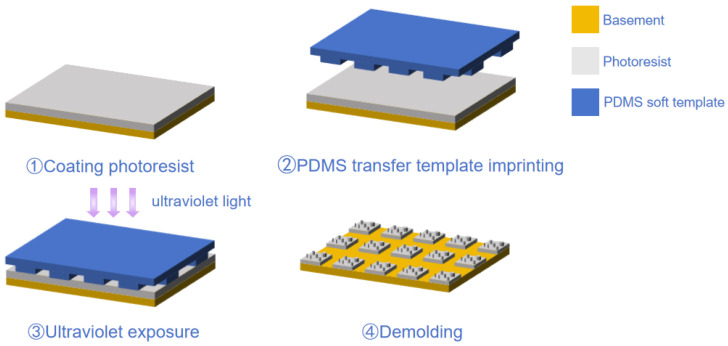
Nanoimprint Lithography Process for Micronano-Composite Layered Structures.

In this study, ultraviolet nanoimprint lithography (UV-NIL) (machine model: EVG 610 UV-Nanoimprint Lithography System, EV Group, Sankt Florian am Inn, Austria) combined with a PDMS soft-template transfer process was employed to achieve high-precision fabrication of the micro–nano-laminated structure. The fabrication process is illustrated in Figure 7.

First, 120 g of PDMS mixture was prepared by blending the prepolymer and curing agent at a 1:1 mass ratio. The mixture was stirred evenly for 3 min until white foaming appeared, indicating complete mixing. It was then degassed under vacuum for 40 min until clear and transparent, signifying that all bubbles had been removed. If bubbles were generated during pouring, an additional degassing step was conducted to ensure a bubble-free solution. During the curing of the soft mold, a level gauge was used to verify horizontal alignment of the mold, ensuring uniform coverage of the PDMS mixture across the master surface without air entrapment. The mold was cured on a heating stage at 90 °C for 1 h, while keeping the master temperature below 100 °C to prevent thermal damage. After curing, the soft mold was obtained by a peeling-based demolding method.

For the imprinting process, a negative-tone epoxy-based photoresist (Light Steering Imprint Glue 1040, micro resist technology GmbH, Berlin, Germany) was employed. Based on spin-coating optimization, the mass ratio of imprint resin to thinner was set to 5:1. The coating was performed in two steps: a low-speed spin at 600 rpm for 30 s followed by a high-speed spin at 3500 rpm for 60 s. The substrate was prebaked at 90 °C for 60 s and post-baked at 110 °C for 180 s. The imprinting temperature was set to 80 °C to enhance resin fluidity and shorten filling time. The contact speed between the substrate and the PDMS mold was maintained at 0.5 mm/s to minimize trapped air and prevent defects. An imprint pressure of 15 kPa was applied to ensure complete filling of the micro–nano-features while avoiding excessive deformation of the mold. The resin filling time was 60 s, followed by UV exposure at an intensity of 0.012 mW/cm^2^ for 60 s. After imprinting, demolding was carried out immediately at the same elevated temperature to prevent resin shrinkage during cooling, which could otherwise damage the transferred pattern. A peeling-type demolding approach was adopted.

In summary, by systematically optimizing key parameters such as imprinting pressure, temperature, UV exposure energy, and the mechanical properties of the soft mold, this study achieved high-fidelity and reproducible replication of micro–nano-hybrid structures. The proposed process ensures structural integrity and surface quality while significantly enhancing fabrication precision and stability, providing a feasible route for scalable manufacturing of multiscale optical and functional microstructures.

## 4. Results and Discussion

### 4.1. SEM Characterization Results of the Master Mold

**Figure 8:**
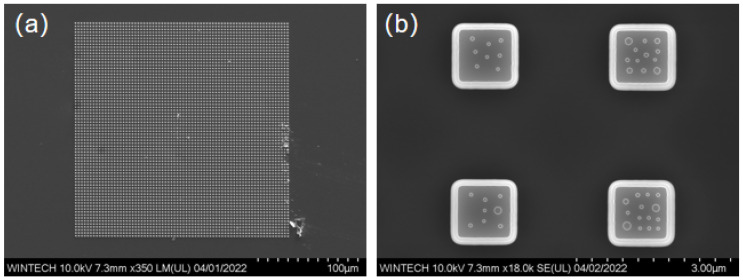
SEM images showing the detailed arrangement of the micron-scale platforms and the nanopillar arrays on their tops. (**a**) Overall morphology of the unit array. (**b**) Morphology of the micron platform and the nanopillars.

The fabricated micronano-composite layered structure is characterized using SEM to examine two-dimensional planar morphology and overall structural distribution. Figure 8a displays the overall morphology of the 72 × 72 unit array, where the bottom-layer micron platform arrangement is regular, and the unit spacing matches the design, demonstrating excellent processing consistency and size control. Figure 8b illustrates the detailed structure of the micron platform and the arrangement of the top-layer nanopillars, which are distributed irregularly across the platform surface. The size and spacing of the nanopillars are consistent with the design, featuring sharp edges and no significant defects, indicating high-quality contour replication achieved through EBL and ICP processes.

**Figure 9:**
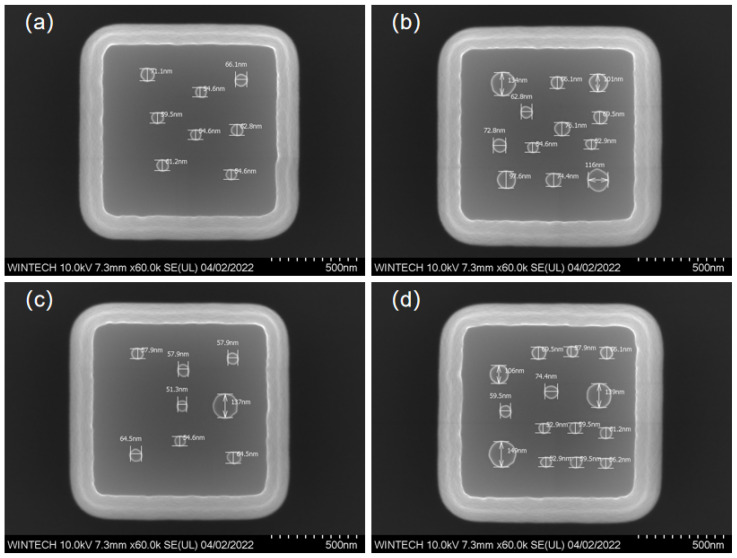
SEM characterization results of the nanopillar morphology and dimensions. (**a**) Morphology and dimensional image of the first nanopillar arrangement type. (**b**) Morphology and dimensional image of the second nanopillar arrangement type. (**c**) Morphology and dimensional image of the third nanopillar arrangement type. (**d**) Morphology and dimensional image of the fourth nanopillar arrangement type.

Figure 9 displays the morphology and size characterization results of four different nanopillar arrangement schemes. The nanopillar morphology is clear, with no noticeable collapse, and the structural integrity is high. The distribution, size, and arrangement of the nanopillars are consistent with the design, indicating that the multi-step process combining EBL and ICP achieves high processing precision and stability at the micronano-scale.

**Figure 10:**
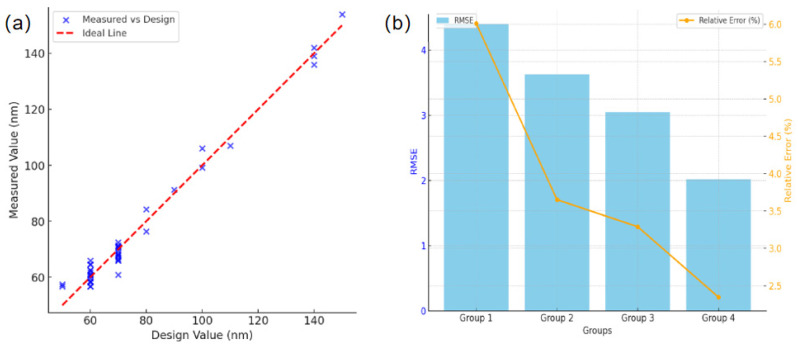
Deviation analysis of nanopillar dimensions. (**a**) Scatter plot of dimensional deviations. (**b**) Bar chart of the root mean square error (RMSE) of dimensional deviations.

Figure 10a,b analyze the size deviation of the nanopillars using scatter plots and root mean square error (RMSE). Most of the size data points are clustered near the ideal consistency line, with slight deviations mainly observed in the smaller nanopillars (50–70 nm in diameter), which may result from exposure dose control and proximity effects. Larger structures (100–150 nm) exhibit better performance. The RMSE and relative errors remain low, further confirming the processing accuracy and stability.

### 4.2. AFM Characterization Results of the Master Mold

**Figure 11:**
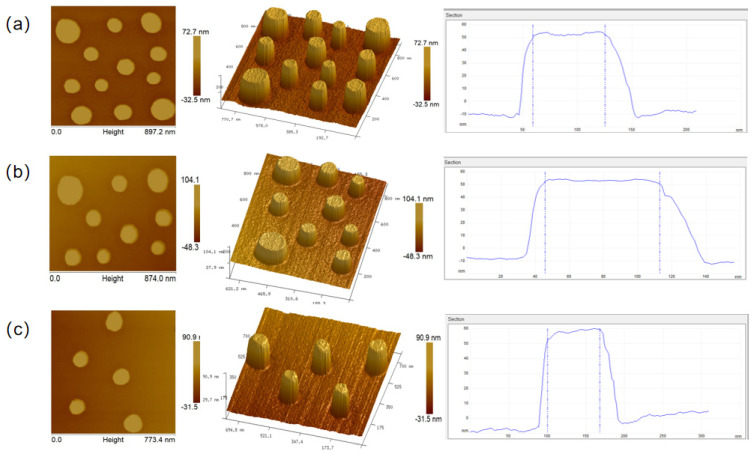
AFM characterization results of nanopillar morphology and dimensions. (**a**) Morphology and dimensional image at position a. (**b**) Morphology and dimensional image at position b. (**c**) Morphology and dimensional image at position c.

The final micronano-composite layered structure is further characterized using AFM to obtain three-dimensional morphological information, such as height profiles. An intelligent probe with a tip curvature radius of approximately 15 nm is employed to scan the micron steps and nanopillars with high precision. Figure 11a–c display the results of the AFM characterization, where the nanopillars on the micron platform are shown with high resolution. The nanopillar distribution is consistent with the design, and the surface morphology is relatively flat, indicating the structural stability of the nanopillars. The height profiles and three-dimensional morphology maps reveal that the nanopillars exhibit good uniformity, and the structure remains stable.

### 4.3. Probe Convolution Effect Correction Methods

**Figure 12:**
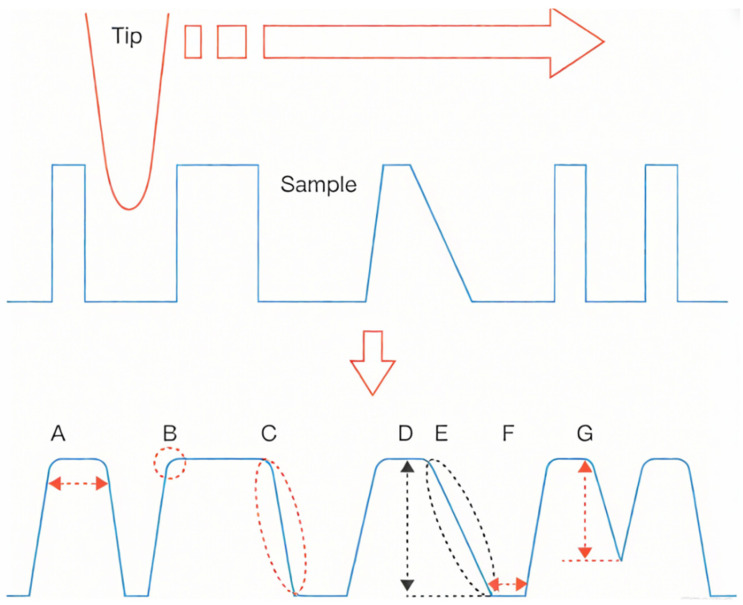
Illustration of the AFM tip-convolution effect. (**A**–**C**) Schematics showing the magnification effect. (**D**–**G**) Schematics showing the tip passivation effect.

However, the characteristic dimensions of the nanopillars exhibited significant deviations from their designed values, primarily because atomic force microscopy (AFM) imaging relies on a microcantilever tip with an extremely small radius of curvature to probe the sample surface. The acquired topography represents a convolution between the AFM tip geometry and the actual surface morphology of the sample. For convex or elevated features, this convolution effect produces an apparent magnification of the structure, as illustrated in Figure 12A–C, where the surface steepness increases and the originally vertical sidewalls appear curved in the planar view. Because AFM operates in contact mode, repeated scanning can lead to tip or sample passivation, thereby exacerbating the convolution effect. As shown in Figure 12D–G, such tip degradation not only amplifies the apparent magnification but also alters the measured height and lateral dimensions of the nanostructures [53,54,55].

**Figure 13:**
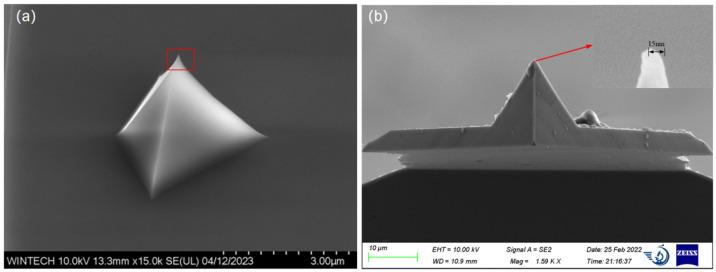
Dimensions of the AFM probe. (**a**) Morphology image of the AF probe. (**b**) Dimensional image of the AFM probe.

Therefore, to obtain more accurate characteristic dimensions of the nanopillars, the lateral size measured at the nanopillar top was corrected by subtracting the radius of curvature of the AFM probe tip. The three-dimensional morphology and curvature radius of the AFM probe tip are shown in Figure 13, indicating a tip radius of 15 nm. This reveals a pronounced measurement deviation caused by the tip–sample convolution effect. Accordingly, the measured lateral dimensions of the nanostructures were quantitatively corrected based on the convolution effect of the AFM probe, and the calculated and corrected dimensional data are summarized in Table 2 (corresponding to Figure 11a–c). The correction results demonstrate that all measured characteristic dimensions of the nanostructures are consistent with the designed values, with dimensional fluctuations confined within ±5 nm, thereby confirming the high precision and reproducibility of the fabrication process.

Scanning electron microscopy (SEM) possesses outstanding morphological imaging capabilities, providing macroscopic information on the surface structural distribution of samples. In contrast, atomic force microscopy (AFM) offers precise three-dimensional topographical information of the surface. By incorporating the AFM tip–convolution correction approach, geometric distortions induced by the probe shape can be effectively compensated, thereby substantially improving the accuracy of quantitative surface characterization. The integrated characterization combining SEM and AFM, along with the AFM tip–convolution correction, enables the simultaneous realization of high precision and reliability in morphological reconstruction and dimensional metrology. This integrated approach achieves full-scale, multidimensional information fusion of micro–nano-composite structures, overcoming the inherent limitations of single-technique characterization. It faithfully reconstructs the true dimensions, spatial distribution, and microscopic morphology of the samples, thereby ensuring both the accuracy and reliability of micro–nano-structural characterization.

### 4.4. SEM Characterization Results of Nanoimprinted Structures

In this study, the lateral characteristic dimensions of the nanostructures were considered the primary criterion for evaluating the fabrication process. Compared with AFM, SEM offers a larger field of view, clearer interfacial morphology, and superior efficiency in revealing the overall structural continuity. Consequently, the lateral characteristic dimensions of the nanostructures measured by SEM are regarded as more accurate. Therefore, SEM was employed to characterize the micro–nano-composite multilayer structures after nanoimprinting.

**Figure 14:**
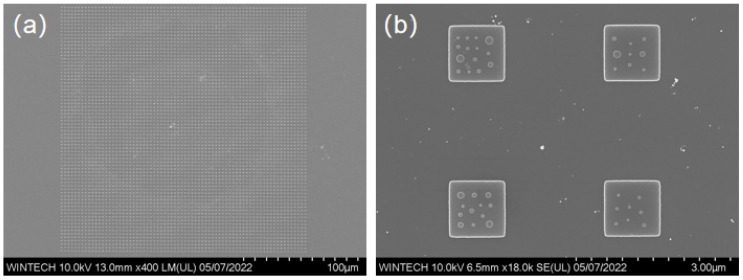
SEM images of the nanoimprinted micro-platform array and the planar arrangement of nanopillars on the top surface. (**a**) Overall morphology of the unit array. (**b**) Morphology of the micro-platforms and nanopillars.

Figure 14a illustrates the overall morphology of a 72 × 72 unit array, where the bottom-layer micron platform is regular, and the structure demonstrates good uniformity and consistency. The unit spacing matches the master design, demonstrating that nanoimprint lithography achieves relatively uniform pattern transfer. Figure 14b displays the detailed arrangement of the top nanopillars, which are distributed irregularly across the platform surface, with good consistency in size and spacing, and no obvious defects.

**Figure 15:**
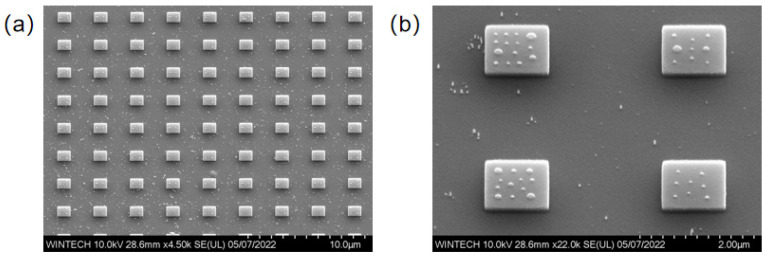
SEM images of the nanoimprinted micro-platform array and the three-dimensional arrangement of nanopillars on the top surface. (**a**) Overall morphology of the unit array. (**b**) Morphology of the micro-platforms and nanopillars.

**Figure 16:**
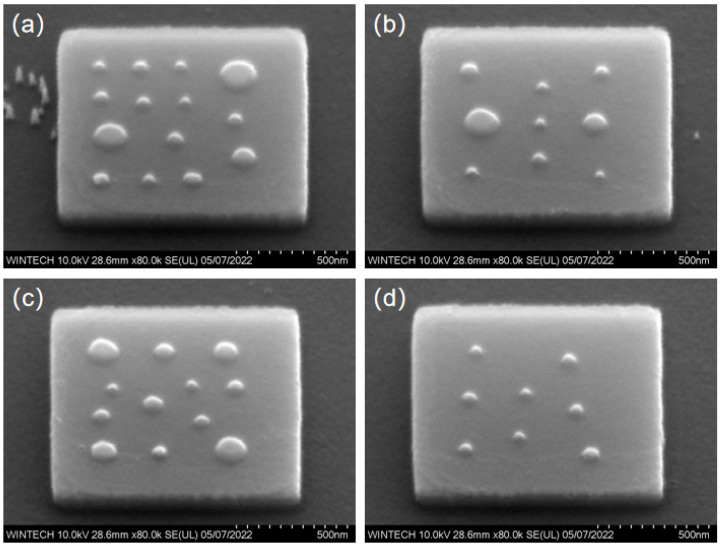
SEM images of the nanopillar morphology on the top surface after nanoimprinting. (**a**) Morphology of the first nanopillar arrangement type. (**b**) Morphology of the second nanopillar arrangement type. (**c**) Morphology of the third nanopillar arrangement type. (**d**) Morphology of the fourth nanopillar arrangement type.

Figure 15 illustrates the morphology of individual micron platform units. The micro-platforms are smooth, with clear edges and regular contours. The sizes are uniform, and no obvious defects are visible. Figure 16 displays the morphology details of four different nanopillar arrangement schemes. The nanopillar morphology is consistent, the distribution is uniform, and no obvious tilting or collapse is observed. Only a few nanopillars exhibit size discrepancies.

**Figure 17:**
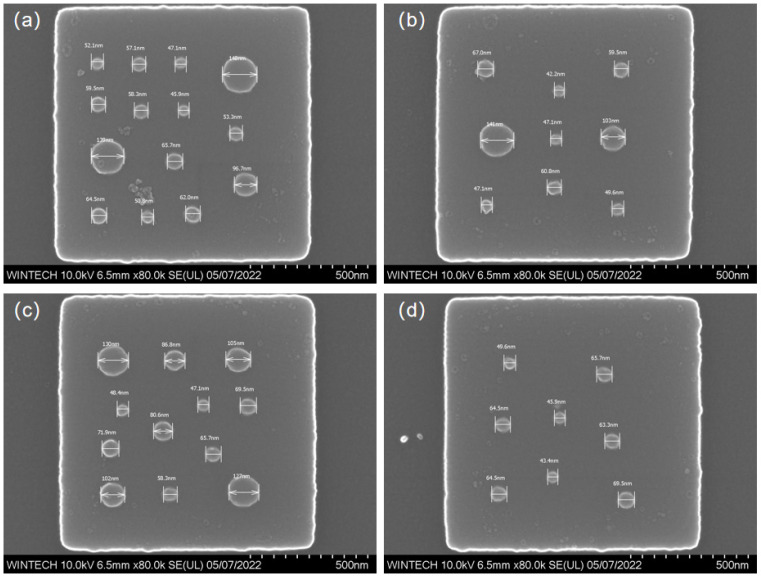
SEM characterization results of nanopillar morphology and dimensions after nanoimprinting. (**a**) Morphology and dimensional image of the first nanopillar arrangement type. (**b**) Morphology and dimensional image of the second nanopillar arrangement type. (**c**) Morphology and dimensional image of the third nanopillar arrangement type. (**d**) Morphology and dimensional image of the fourth nanopillar arrangement type.

Figure 17a–d displays the morphology and size characterization results of four different nanopillar arrangement schemes. The nanopillars’ distribution, size, and arrangement are consistent with the design, confirming that the NIL process can achieve high-precision replication and fabrication at the nanoscale.

**Figure 18:**
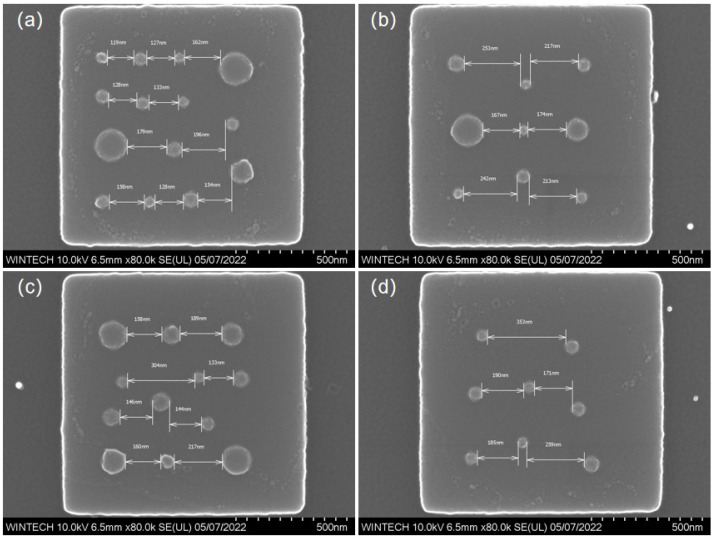
SEM characterization results of nanopillar spacing on the top surface after nanoimprinting. (**a**) Morphology and spacing dimensional image of the first nanopillar arrangement type. (**b**) Morphology and spacing dimensional image of the second nanopillar arrangement type. (**c**) Morphology and spacing dimensional image of the third nanopillar arrangement type. (**d**) Morphology and spacing dimensional image of the fourth nanopillar arrangement type.

Figure 18a–d illustrate the lateral spacing of the nanopillars in four different arrangement schemes. The nanopillars’ distribution and spacing are consistent with the design, with no significant deviation from the expected values. The results demonstrate that the multi-step process combining EBL, ICP, and NIL can achieve high-precision manufacturing of micronano-composite layered structures, effectively addressing alignment issues due to scale effects during processing, and improving production efficiency.

## 5. Conclusions

This work innovatively proposes a high-precision fabrication strategy for micro–nano-composite multilayer structures composed of micron-scale platforms and nanopillar arrays, achieved through a hierarchical multi-step synergistic process integrating electron-beam lithography (EBL), inductively coupled plasma (ICP) etching, and nanoimprint lithography (NIL). Experimental results demonstrate that the fabricated micro-platform structures exhibit excellent uniformity and structural stability, while the nanopillar arrays display well-defined morphology, uniform spatial distribution, and negligible alignment deviation. This approach effectively mitigates the thermal expansion mismatch between heterogeneous material layers and minimizes alignment errors originating from equipment precision limitations. Moreover, it reduces fabrication costs and enhances overall process throughput.

Furthermore, an integrated SEM–AFM characterization methodology incorporating AFM tip–convolution correction was established. After correction, the average lateral dimension of the nanopillars was 52.541 nm, which closely matched the designed value. This realization enables full-scale and multidimensional quantitative characterization of micro–nano-composite structures, ensuring the precision and fidelity of both dimensional and morphological measurements.

The present study not only offers a novel paradigm for the fabrication of multilayer composite micro–nano-structures but also elucidates the intrinsic measurement deviations between SEM and AFM, providing an essential theoretical foundation and technical support for the development of advanced photonic devices. This advancement is expected to promote emerging applications in high-sensitivity molecular detection, super-resolution optical imaging, and other frontier nanophotonic technologies.

## Figures and Tables

**Table 1:** Fabrication Process Parameters.

Processing Step	Parameter	Value
Resist Coating	Low-speed RPM	500 rpm
	High-speed RPM	4000 rpm
	Bake Temperature	180 °C
Bottom Layer Exposure (EBL)	Voltage	20 kV
	Beam Current	50 pA
	Exposure Dose	250 μC/cm^2^
Top Layer Exposure (EBL)	Beam Current	30 pA
	Exposure Dose	150 μC/cm^2^
ICP Etching (Bottom Layer)	SF_6_ Flow Rate	50 sccm
	C_4_F_8_ Flow Rate	30 sccm
	Chamber Pressure	20 mTorr
ICP Etching (Top Layer)	SF_6_ Flow Rate	30 sccm
	C_4_F_8_ Flow Rate	10 sccm
	Chamber Pressure	15 mTorr

**Table 2:** Corrected Nanopillar Dimensions.

Test Item	Test Result Description				
AFM	Lateral Feature Size Measurement of Nanostructures (nm)		Probe Tip Curvature Radius (nm)	Corrected Nanopillar Dimensions (nm)	Average Corrected Dimension (nm)
	Position a	65.884	15	51.884	52.541
	Position b	65.893		51.893	
	Position c	67.845		53.845	

## Data Availability

The authors confirm that the data supporting the findings of this study are available within the article.

## Abbreviations

The following abbreviations are used in this manuscript:

EBL	Electron beam lithography
ICP	Inductively coupled plasma
NIL	Ultraviolet nanoimprint lithography
SEM	Scanning electron microscopy
AFM	Atomic force microscopy
PECVD	Plasma-enhanced chemical vapor deposition
IPA	Isopropanol
MIBK	Methyl isobutyl ketone
RMSE	Root mean square error
